# What are the beliefs of pediatricians and dietitians regarding complementary food introduction to prevent allergy?

**DOI:** 10.1186/1710-1492-8-3

**Published:** 2012-03-21

**Authors:** Sara Leo, John Dean, Edmond S Chan

**Affiliations:** 1 BC Children's Hospital, Department of Pediatrics, Division of Allergy, Room 1C31B - 4480 Oak Street, Vancouver, BC V6H 3 V4, Canada

**Keywords:** Food allergy, Children, Survey, Prevention, Dietary advice

## Abstract

**Background:**

The timing of complementary food introduction is controversial. Providing information on the timing of dietary introduction is crucial to the primary prevention of food allergy. The American Academy of Pediatrics offers dietary recommendations that were updated in 2008.

**Objective:**

Identify the recommendations that general pediatricians and registered dietitians provide to parents and delineate any differences in counselling.

**Methods:**

A 9-item survey was distributed to pediatricians and dietitians online and by mail. Information on practitioner type, gender, length of practice and specific recommendations regarding complementary food introduction and exposure was collected.

**Results:**

181 surveys were returned with a 54% response rate from pediatricians. It was not possible to calculate a meaningful dietitian response rate due to overlapping email databases. 52.5% of all respondents were pediatricians and 45.9% were dietitians. The majority of pediatricians and dietitians advise mothers that peanut abstinence during pregnancy and lactation is unnecessary. Dietitians were more likely to counsel mothers to breastfeed their infants to prevent development of atopic dermatitis than pediatricians. Hydrolyzed formulas for infants at risk of developing allergy were the top choice of formula amongst both practitioners. For food allergy prevention, pediatricians were more likely to recommend delayed introduction of peanut and egg, while most dietitians recommended no delay in allergenic food introduction.

**Conclusions:**

In the prophylaxis of food allergy, pediatricians are less aware than dietitians of the current recommendation that there is no benefit in delaying allergenic food introduction beyond 4 to 6 months. More dietitians than pediatricians believe that breastfeeding decreases the risk of atopic dermatitis. Practitioners may benefit from increased awareness of current guidelines.

## Background

Food allergy is a hypersensitivity reaction to food allergens initiated by the immune system [1,2]. IgE-mediated food allergy (type I), forms the bulk of food-induced allergic responses and results in elevated allergen-specific serum IgE antibodies. It is not clear how the gut mucosal immune system is oriented toward sensitization versus immune tolerance when exposed to dietary antigens [3]. Food allergy is prevalent, affecting 1 - 10 % of children worldwide [4,5].

Most food-induced allergic reactions occur at first known oral exposure [6]. Hence, the timing of the first introduction of complementary foods (food other than breast milk or infant formula) has been of great interest. Different foods are allergenic in different age groups.

Two diseases that commonly coexist with food allergy are atopic dermatitis and asthma. Eczema is often the first manifestation of atopic disease, manifesting at 6 - 12 months old. Those with systemic allergic disease often have more than one food allergy, as well as asthma and allergic rhinosinusitis [6].

The theory that early oral exposure to food allergens can cause the developing immune system to produce specific IgE has led to delay in the weaning of breast milk, which in turn delays complementary food introduction, to prevent allergy development [6]. However, randomized controlled trials of food allergen elimination from the infant diet or from the diet of pregnant or lactating mothers have not shown reductions in risk of developing food allergy [6-8]. The 2000 AAP statement [9] has been replaced by the 2008 AAP statement [1] with updated recommendations on the timing of complementary food introduction (Table 1).

**Table 1:** Comparing the 2000 and 2008 AAP Statements on the timing of introduction of complementary foods

**2000 AAP Statement **[9]	**2008 AAP Statement **[1]
No maternal dietary restrictions during pregnancy are necessary with the possible exception of excluding peanuts.	Lack of evidence that maternal dietary restrictions during pregnancy play a significant role in the prevention of atopic disease in infants.
Infants at high risk of allergy should be breastfed or given a hypoallergenic formula.	1. Infants at high risk of allergy that are exclusively breastfed, on extensively hydrolyzed or partially hydrolyzed formula, for at least 4 months have a decreased incidence of atopic dermatitis. 2. Extensively hydrolyzed formulas may be more effective than partially hydrolyzed in the prevention of atopic disease. 3. Amino-acid base formulas for atopy prevention have not been studied. 4. No convincing evidence for the use of soy-based infant formula for the purpose of allergy prevention.
	
	Infants at high risk of allergy that are exclusively breastfed for at least 4 months have a decreased incidence of cow milk allergy in the first 2 years of life.
	
	Infants at high risk of allergy that are exclusively breastfed for at least 3 months are better protected against wheezing in early life; no evidence that this protects against allergic asthma after 6 years old.

Mothers should eliminate peanuts and tree nuts (almonds, walnuts, etc) while nursing.	Lack of evidence that maternal dietary restrictions while nursing play a significant role in the prevention of atopic disease in infants.
	
Mothers should consider eliminating eggs, cow's milk, fish from their diets while nursing.	

Infants at high risk of allergy should have dairy products delayed until 1 year, eggs until 2 years and peanuts, nuts and fish until 3 years (no evidence presented).	Solid foods should not be introduced before 4 - 6 months of age, but there is no current convincing evidence that delaying their introduction beyond this period has a significant protective effect on the development of atopic disease. This includes the delay of foods considered to be highly allergic such as fish, eggs, and foods containing peanut protein. For infants older than 4 to 6 months old, there is insufficient data to support a protective effect of any dietary intervention for the development of atopic disease.

We wanted to identify recommendations practitioners are currently giving about complementary food introduction. We are unaware of any other studies in the literature which seek to identify how closely current recommendations on complementary food introduction are being followed.

We chose to focus on general pediatricians and dietitians who were likely to regularly care for infants and young children, exploring: How do general pediatricians and dietitians currently counsel parents on the introduction of foods? Is there a difference between advice offered by general pediatricians and registered dietitians (RD)? If knowledge deficits are identified, further education could address the deficits specifically.

## Methods

This was a cross-sectional study of general pediatricians and registered dietitians in British Columbia (BC). The study was approved by the BC Children's and Women's Behavioural Research Ethics Board.

### Survey creation

A 9-item survey collected information on practitioner type, gender, length of practice and specific recommendations made regarding complementary food introduction and exposure. The survey was designed in online and mail versions. Survey questions were based on recommendations in the 2008 AAP statement on introduction of complementary foods (Additional file 1).

### Survey distribution

The survey was mailed to BC general pediatricians with addresses obtained from the College of Physicians and Surgeons of BC. Pediatricians whose practices were dedicated to sub-specialties were not surveyed. It was only possible to access the dietitians online (no published mailing addresses available), so an email with an online link to the survey (RedCAP Survey engine, Child and Family Research Institute) was distributed by a BC Children's Hospital dietitian to three dietitian online mailing lists. The surveys were distributed and collected for all groups from June to October 2010.

### Statistical analyses

Statistical analysis was performed by our institution's statistician using SPSS (version 18). Frequencies were presented and categorical variables were evaluated by the chi square test. A p-value < 0.05 was considered statistically significant.

## Results

### Survey response rate

Surveys were mailed to 176 general pediatricians. An email survey link was sent to 3 dietitian mailing lists (consisting of registered dietitians and interns), with approximately 1300 recipients. Due to overlap between the same names on different dietician mailing lists and reaching more than registered dietitians (e.g. students received the survey as well), it was not possible to calculate a meaningful dietitian response rate.

A total of 181 surveys were returned with 47.5% collected online and 52.5% collected by mail. 92.8% of surveys returned were filled completely. 52.5% of respondents were pediatricians and 45.9% were dietitians. The pediatrician response rate was 54%.

### Demographic information

45.3% were community pediatricians, 6.1% were academic pediatricians, and 1.1% were both academic and community pediatricians. 28.2% were community dietitians and 17.7% were hospital dietitians (Table 2). Hence, the largest group of respondents were community pediatricians. 22.7% of respondents were male and 75.7% were female. The majority of pediatricians and dietitians had spent greater than fifteen years in practice (42.5%); 21.5% had spent less than five years in practice, 16.0% had spent five to ten years in practice and 18.2% had spent ten to fifteen years in practice.

**Table 2:** Demographic information of pediatricians and dietitians surveyed

Occupation	Number of respondents	0 - 5 y	5 - 10 y	10 - 15 y	> 15 y	Male	Female
**Community Pediatrician**	82	14	12	19	37	34	48

**Academic Pediatrician**	11	1	6	0	4	2	9

**Community dietitian**	51	10	5	11	25	3	48

**Hospital based dietitian**	32	13	6	3	10	1	31

**Both community and academic pediatrician**	2	1	0	0	1	1	1

**Unknown (all data missing)**	3	-	-	-	-	-	-

**Total**	181	39	29	33	77	41	137

Most community pediatricians (54.9%) had been in practice for less than fifteen years and 58.5% were female. Most academic pediatricians (54.5%) had been in practice five to ten years and 81.8% were female. Nearly half (49%) of community dietitians had been in practice greater than fifteen years and 94.1% were female. The majority of hospital dietitians (40.6%) had been in practice less than 5 years and 96.9% were female.

### Recommendations to mothers regarding abstinence from allergenic foods

#### Abstinence from peanuts during pregnancy

94.5% of pediatricians and 95.2% of dietitians did not recommend abstinence from peanuts during pregnancy (p = 0.558). There were no differences when the data was analyzed by length of practice or gender. Some respondents who didn't answer this question stated they rarely see pregnant mothers.

#### Abstinence from peanuts while breastfeeding

The majority of pediatricians and dietitians, 89.1% and 88.0% respectively, did not recommend mothers abstain from eating peanuts while breastfeeding (p = 0.827). There was no difference between how community (88.2%) and hospital based dietitians (87.5%) counselled based on length of practice and gender. Although not statistically significant, when pediatricians were analyzed by gender, 96.5% of female pediatricians compared with 77.1% of male pediatricians recommended against peanut abstinence while breastfeeding; when analyzed by length of practice, 97.0% of those less than ten years in practice compared with 84.7% of those greater than ten years in practice recommended against peanut abstinence while breastfeeding.

#### Avoidance of allergenic foods during breastfeeding

The majority of pediatricians (59.1%) did not advise mothers to avoid allergenic foods during breastfeeding. 3.2% of pediatricians would advise mothers of all infants and37.6% would advise only mothers of infants at high risk of allergic disease to avoid allergenic foods during lactation. Similarly, the majority of dietitians (64.6%) did not advise mothers to avoid allergenic foods while breastfeeding. 2.4% of dietitians would advise mothers of all infants and 32.9% would advise only mothers of high risk infants to avoid allergenic foods during breastfeeding. Two respondents indicated that they do not counsel mothers to avoid allergenic foods during breastfeeding "unless the child has a milk allergy", and one "unless the baby has symptoms".

### Recommendations for breastfeeding to prevent atopic dermatitis

66.3% of pediatricians and 85.4% of dietitians advised mothers to breastfeed for the first four months of life to prevent atopic dermatitis (p = 0.004). When analyzed further, 94.0% of community dietitians, 71.9% of hospital based dietitians, 67.9% of community pediatricians and 54.5% of academic pediatricians advised mothers to breastfeed in order to prevent atopic dermatitis. A number of respondents highlighted they do not specifically advise mothers to breastfeed to prevent atopic dermatitis; rather they counsel mothers to breastfeed for other health benefits of breast milk.

### Recommended formulas for an infant with high risk of developing allergy

**Figure 1:**
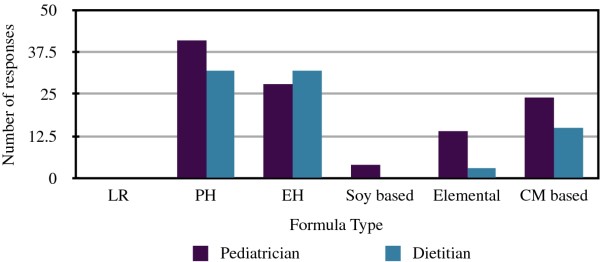
**Recommended formulas for infants at risk of allergy**. LR, lactosed reduced; PH, partially hydrolyzed; EH, extensively hydrolyzed; CM, cow's milk.

This question (Figure 1) was answered in a multiple response fashion by pediatricians (mail survey), hence the number of responses exceeds the number of pediatricians. Dietitians picked the single best answer as the online survey only permitted one answer. Most pediatricians recommended a partially hydrolyzed formula (41/111), followed by extensively hydrolyzed (28/111) formula. The recommendation for cow's milk based formula was almost equal to extensively hydrolyzed. Dietitians recommended the hydrolyzed formulas equally, followed by cow's milk based formula. No dietitians recommended soy formula and no practitioners recommended lactose reduced formula. One written comment suggested elemental formula as one of the options.

### Foods recommended for delayed introduction to prevent development of allergy

**Figure 2:**
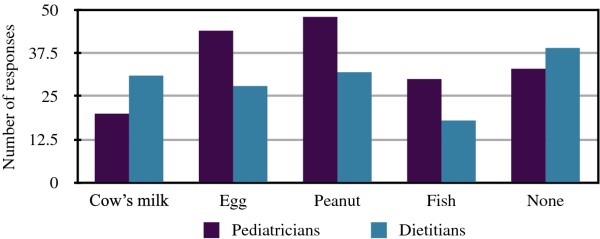
**Recommended foods for delayed introduction**.

Pediatricians were most likely to recommend avoidance of peanut and egg (Figure 2). They were less likely to recommend delay of fish or no delay of allergenic foods at all. 20/93 recommended delaying cow's milk. Amongst dietitians, the greatest number would recommend no delay of potentially allergenic foods, followed by cow's milk and peanut, then egg and fish.

## Discussion

With the exception of breastfeeding advice and delay of allergenic foods, pediatricians and dietitians of BC generally agree in their advice and adhere to the 2008 AAP guidelines.

We expected pediatricians would recommend breastfeeding as often as dietitians to prevent atopic dermatitis, but pediatricians recommended it less often (statistically significant). The 2008 AAP statement states that infants at high risk of allergy who are exclusively breastfed [10,11] for at least 4 months have a decreased incidence of atopic dermatitis [12]; these infants also have decreased incidence of cow's milk allergy in the first 2 years of life [13]. Infants breastfed for at least 3 months are protected against wheezing in early life [11,14]. However, a longitudinal study suggested that breastfeeding may actually increase the risk of atopy and asthma later in life [15]. We speculate that pediatricians responded the way they did because many recommend breastfeeding for the other benefits of breast milk, rather than to specifically prevent atopic dermatitis and wheezing. It would have been useful to collect qualitative feedback to understand the rationale for their response.

We also found a difference in the recommendations given by pediatricians and dietitians regarding the avoidance of allergenic foods. Reflective of the 2000 AAP guidelines, pediatricians were more likely to recommend delay of specific foods. These results suggest both groups, but especially pediatricians, would benefit from further education on the lack of benefit in delaying specific food proteins for infants beyond 4 to 6 months of age. The initial premise behind food avoidance/delay to prevent allergy was twofold; firstly, to decrease the incidence of a severe reaction in younger children and secondly, to prevent early gut exposure which was thought to cause sensitization and a subsequent increase in allergy. However, newer evidence suggests that early introduction of some allergenic foods may actually decrease the risk of atopic disease by promoting tolerance through regulatory T-cell pathways, minimizing the chance of sensitization via the skin^4^. Early and regular introduction of cow's milk formula to supplement breastfeeding may prevent cow's milk allergy [16]. Introduction of cooked egg at 4 to 6 months of age might protect against egg allergy [17]. Early and frequent ingestion of high doses of peanut protein during infancy might induce tolerance and thereby prevent the development of peanut allergy [18,19]. The UK LEAP study looking at early versus delayed peanut protein introduction in 640 high risk infants with the outcome of peanut allergy at age five years is currently in progress [20].

In keeping with the lack of consensus in the literature, most dietitians and pediatricians advised mothers that there was no need to abstain from eating peanuts during pregnancy and breastfeeding to prevent the development of peanut allergy. We found that female pediatricians and pediatricians in practice less than ten years were more likely to recommend against peanut abstinence. Studies on peanut during pregnancy are inconclusive. Recently, a study suggested peanut consumption during pregnancy may increase peanut sensitization at 3 to 15 months of age, but there is a clear difference between peanut sensitization (positive allergy skin or blood test to peanut) and true clinical peanut allergy [21]. Low dose weekly peanut exposure during pregnancy and lactation in a mouse model showed that peanut allergy may be decreased [22]. Another study found that daily peanut consumption during pregnancy could increase risk of childhood wheeze and asthma symptoms [23].

Over half of practitioners appropriately counselled that no mothers need avoid allergenic foods while nursing but a significant number would recommend avoidance for high risk infants, which suggests there could be potential benefit to more education in this area. The 2008 guidelines cite a lack of evidence that maternal dietary restrictions while nursing play a significant role in prevention of atopic disease in infants [13,24,25].

Pediatricians were more likely to recommend a partially hydrolyzed rather than an extensively hydrolyzed formula, while equal numbers of dietitians recommended extensively and partially hydrolyzed formulas for allergy prevention. Some studies suggest that extensively hydrolyzed, partially hydrolyzed and amino acid formulas are equally useful for allergy prevention [26], while others suggest there is a differential effect [27-29]. A recent review suggested infants without a history of eczema in a first-degree relative will receive protective effect from partially hydrolyzed formula, but those infants who have first-degree relatives with eczema should receive extensively hydrolyzed formula [29,30]. The protective effect of hydrolyzed infant formulas on atopic eczema may last until 6 years of age [29].

Recommendation of cow's milk formula was the third most popular choice with both groups. Katz et al. found that the incidence of IgE-mediated cow's milk allergy may be decreased by introducing cow's milk based formula early and regularly to infants (daily supplementation of breastfeeding with cow's milk formula). Infants exposed to cow's milk formula before 14 days of age were less likely to develop cow's milk allergy. Infants that were not regularly exposed to cow's milk protein until four to six months of age were at the greatest risk for developing cow's milk protein allergy [16]. Extending our study to explore whether pediatricians who choose cow's milk formula recommend daily ingestion (versus not giving advice on frequency) would be intriguing, and may illustrate a new approach to recommendations based on frequency of ingestion. No convincing evidence exists for the use of soy-based infant formula for the purpose of allergy prevention [31], and we found that only a small number of pediatricians and no dietitians recommended soy formula.

Our study was limited to surveying dietitians and pediatricians. It would be interesting to poll family physicians who provide the bulk of primary care for Canadian children. The study was limited to British Columbia, Canada and it would be interesting to see opinions elsewhere in the world. A potential confounder in this study was the different mode of survey distribution for the two practitioner groups, with the online survey for dietitians and paper survey for pediatricians. Although the wording was identical, we did not anticipate that many pediatricians would answer in a multi-response fashion to the last two questions that were meant to be single response (dietitians only had the option of single response with the online modality).

## Conclusions

Our study has provided us with data suggesting practitioners may benefit from increased awareness of current guidelines. With new thoughts on the possible benefits of oral tolerance for infants introduced to regular ingestion of specific food proteins without delay, we feel it is most important for the awareness to focus on the lack of benefit of delay. We speculate this need is not unique to British Columbia. An extension of our study is currently underway in eastern Canada.

## Abbreviations

AAP: American Academy of Pediatrics; BC: British Columbia; CM: Cow's milk; EH: Extensively hydrolyzed; LR: Lactose reduced; LEAP: Learning Early About Peanut Allergy; PH: Partially hydrolyzed; RD: Registered Dietitian; UBC: University of British Columbia.

## Competing interests

The authors declare that they have no competing interests.

## Authors' contributions

All authors made substantial contributions to the study conception and design, acquisition of data, analysis and interpretation of data. SL drafted the manuscript. All authors were involved in revising the manuscript critically and have given final approval of the published version. All authors read and approved the final manuscript.

## Supplementary Material

Additional file 1 **Appendix 1: Timing of Complementary Food Introduction**. Click here for file
