# Supplementary material for: What are the beliefs of pediatricians and dietitians regarding complementary food introduction to prevent allergy?
Source: Allergy Asthma Clin Immunol. 2012 Mar 21;8(1):3. doi: 10.1186/1710-1492-8-3 (PMC3337797; doi:10.1186/1710-1492-8-3)
Supplement: Additional file 1 — Appendix 1: Timing of Complementary Food Introduction. [file 1710-1492-8-3-S1.DOC]

TIMING OF COMPLEMENTARY FOOD INTRODUCTION

For each question, please mark the box that applies to you.

1. Occupation:

🞏 Community general pediatrician

🞏 Academic general pediatrician

🞏 Community dietitian

🞏 Hospital based dietitian

1. How long have you been in practice?

🞏 0 - 5 years

🞏 5 – 10 years

🞏 10 – 15 years

🞏 Greater than 15 years

1. Gender:

🞏 Male

🞏 Female

1. Do you advise mothers to abstain from eating peanuts during their pregnancy to prevent the development of peanut allergy?

🞏 Yes

🞏 No

1. Do you advise mothers to abstain from eating peanuts while breastfeeding to prevent the development of peanut allergy?

🞏 Yes

🞏 No

1. Which mothers do you counsel to avoid allergenic foods during b
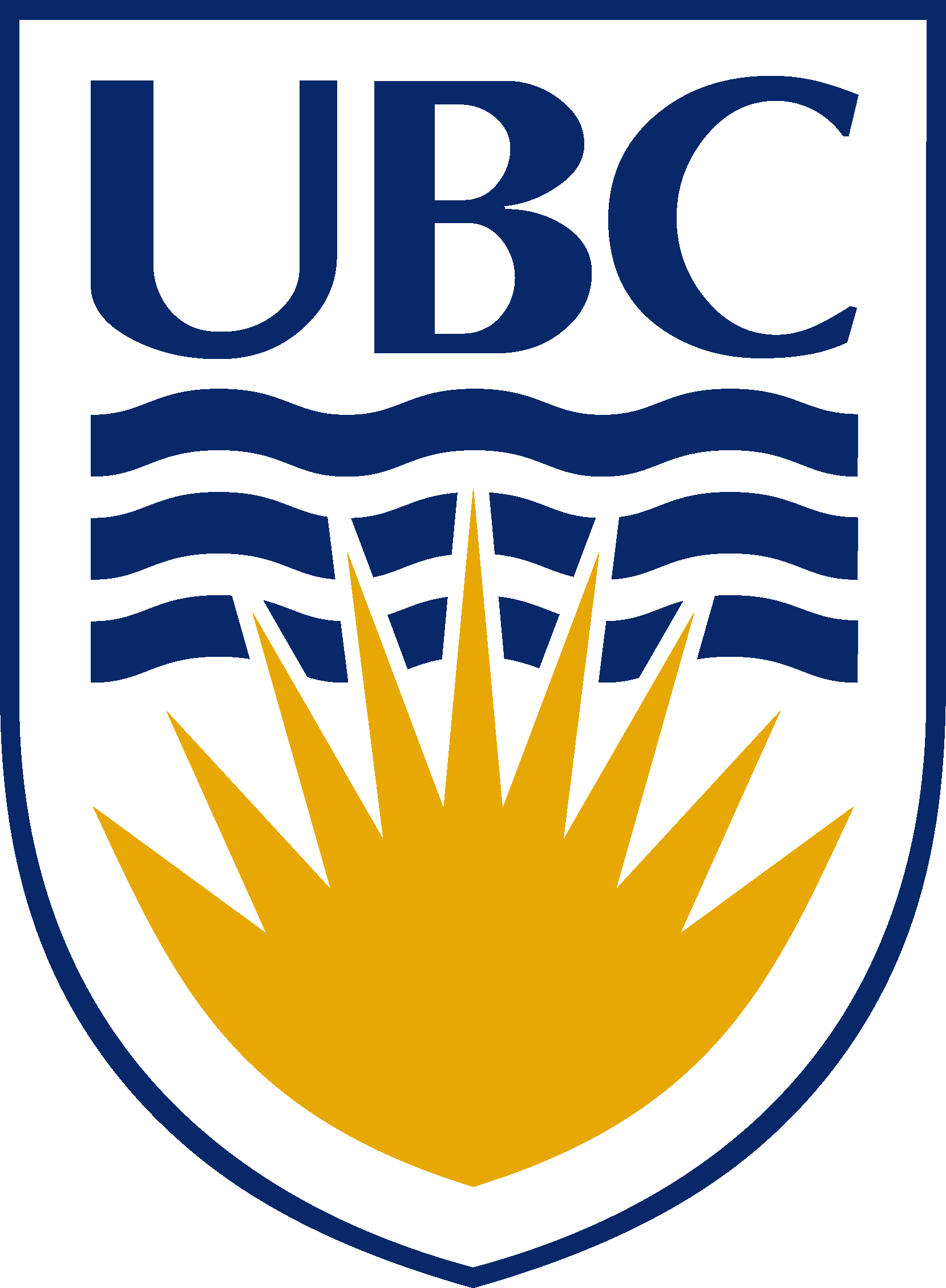

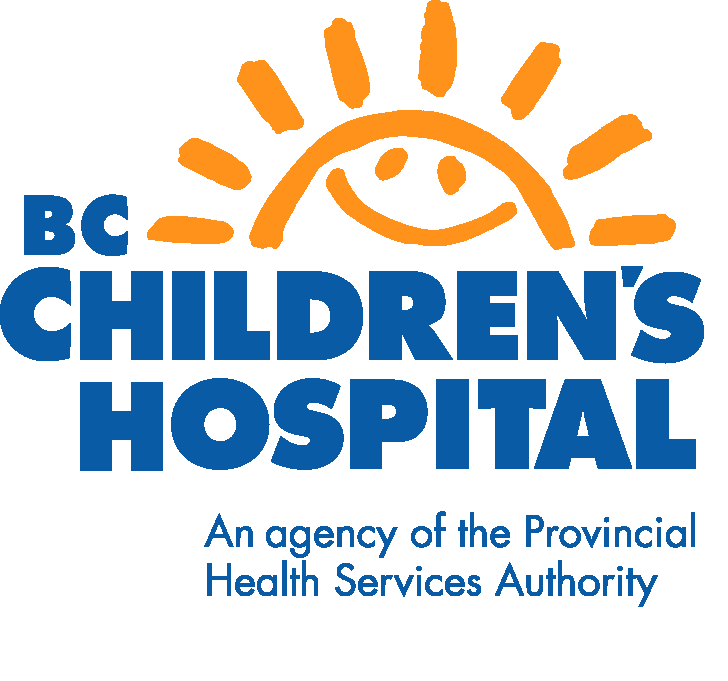

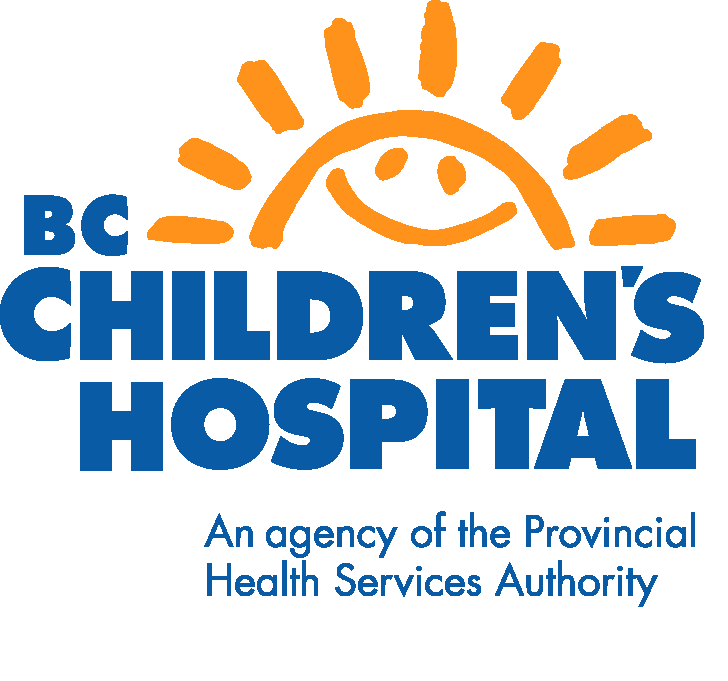
reastfeeding?

🞏 Mothers of all infants

🞏 Mothers of infants with high risk of atopic disease (defined by having at least one first degree

relative with asthma, eczema, allergic rhinitis or food allergy)

🞏 I do not counsel mothers to avoid allergenic foods during breastfeeding

1. Do you advise mothers that they should breastfeed for the first 4 months of life to prevent atopic dermatitis?

🞏 Yes

🞏 No

1. Which of the following types of formulas are you most likely to recommend for an infant with a high risk of developing an allergy?

🞏 Lactose-reduced formula

🞏 Partially hydrolyzed formula

🞏 Extensively hydrolyzed formula

🞏 Soy-based formula

🞏 Elemental formula

🞏 Cow's milk based formula

1. Which of the following foods do you recommend delaying the introduction of to prevent the development of allergy? (Check all that apply)

🞏 Cow’s milk

🞏 Egg

🞏 Peanut

🞏 Fish

🞏 None

Th
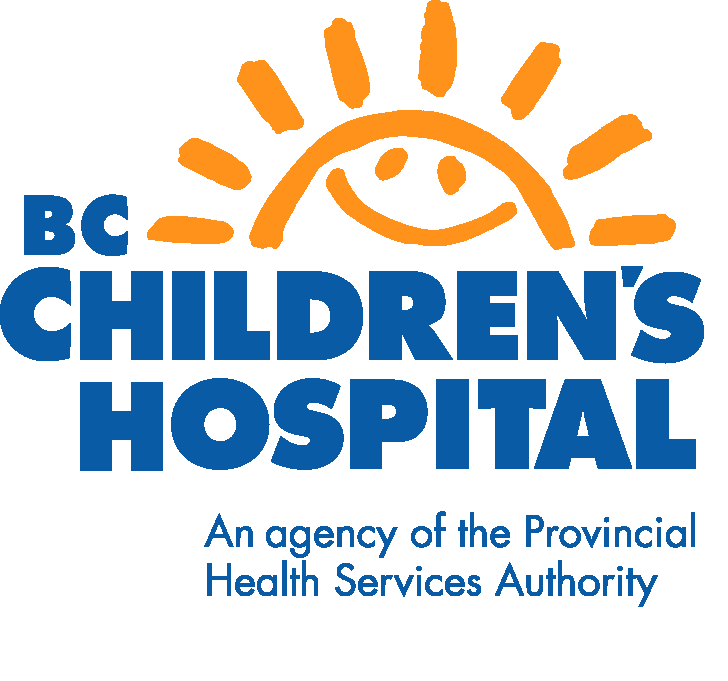

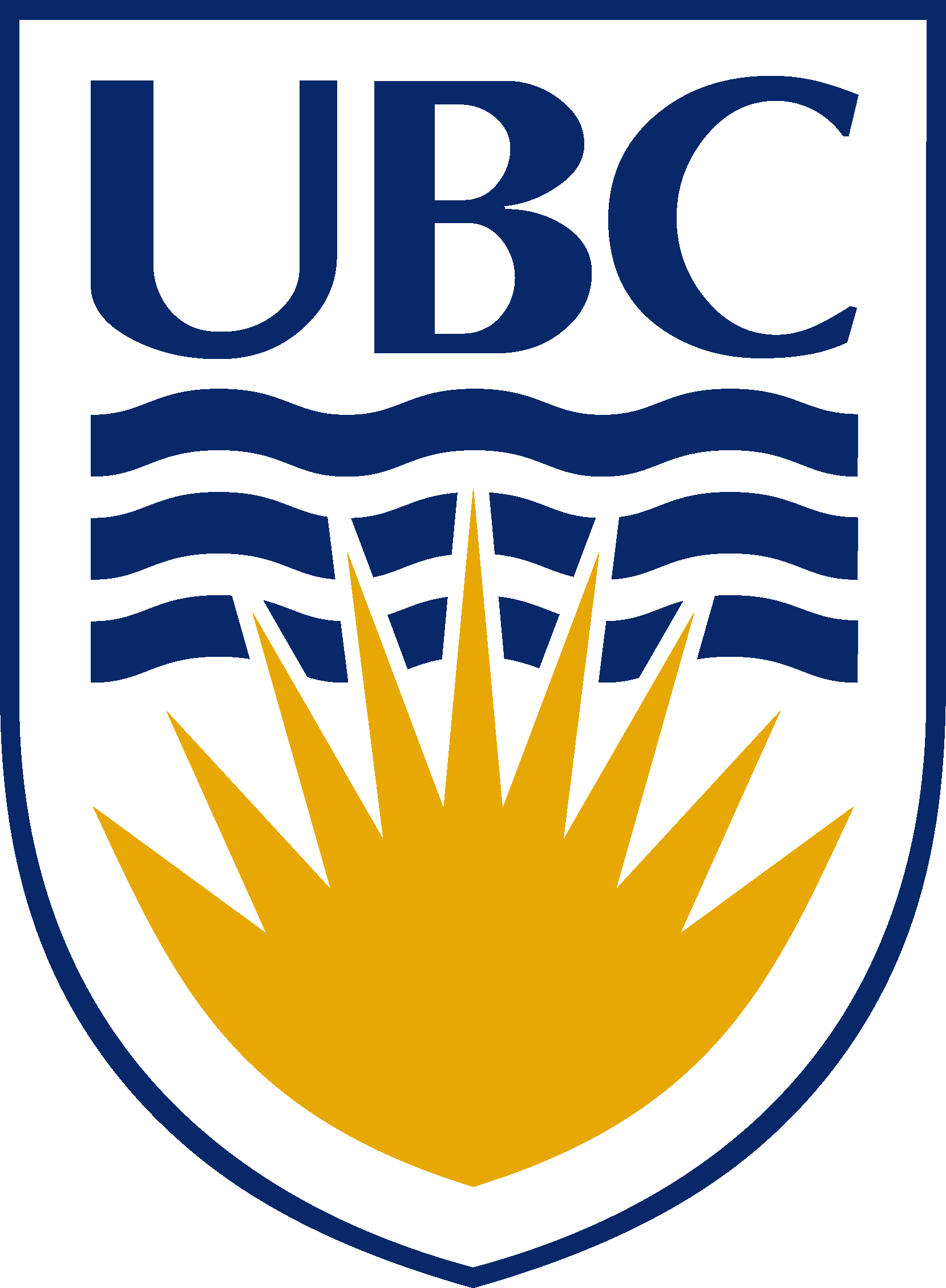
ank you for completing this survey. Please return it in the self-addressed stamped envelope to:

BC Children’s Hospital

Allergy Clinic, Rm. 1C31B

4480 Oak Street

Vancouver, BC

V6H 3V4
